# Clinical and perioperative outcomes of computer-guided versus conventional plan-transfer workflows in orthognathic surgery for skeletal anterior open bite: a randomized controlled trial

**DOI:** 10.3389/fdmed.2026.1923688

**Published:** 2026-09-03

**Authors:** Ammar Abu Shama, Dirar Dibs

**Affiliations:** Department of Oral and Maxillofacial Surgery, Faculty of Dentistry, Al-Quds University, Jerusalem, Palestine

**Keywords:** anterior open bite, computer-guided surgery, orthognathic surgery, perioperative outcomes, virtual surgical planning

## Abstract

**Introduction:**

Skeletal anterior open bite (AOB) often requires orthognathic surgery. This study compared the clinical and perioperative outcomes of computer-guided and conventional plan-transfer workflows following virtual surgical planning.

**Methods:**

Eighteen adults with skeletal AOB underwent virtual surgical planning and were subsequently randomized to computer-guided transfer of the established surgical plan using CAD/CAM guides (*n* = 9) or conventional model-surgery transfer using articulator-mounted casts and occlusal wafers (*n* = 9). Outcomes included intraoperative handling, operative time, blood loss, complications, healing, and AOB correction.

**Results:**

Mean operative time was 6.0 ± 0.5 h versus 6.5 ± 0.6 h (*p* = 0.08), and blood loss was 450 ± 60 versus 520 ± 80 mL (*p* = 0.12) for the computer-guided and conventional groups, respectively. Intraoperative handling was perceived as easier with computer guidance, although this assessment was subjective. No major intraoperative complications occurred. Mean postoperative AOB was 1.33 ± 0.50 mm in both groups (*p* = 1.00).

**Conclusion:**

No statistically significant between-group differences were detected in operative time, blood loss, or postoperative AOB. Computer-guided plan transfer was perceived to facilitate intraoperative handling, although this assessment was subjective. Given the small sample size and limited statistical power, the absence of statistically significant differences should not be interpreted as evidence of equivalence between the workflows. Larger adequately powered studies are warranted.

## 1 Introduction

Skeletal anterior open bite (AOB) is a multifactorial dentofacial deformity characterized by a lack of vertical overlap between the maxillary and mandibular anterior teeth. Anterior open bite is usually seen in association with posterior vertical maxillary excess, a short mandibular ramus, or a combination of both. Adults presenting for treatment of skeletal anterior open bite may experience functional difficulties involving mastication and speech and may present with an elongated facial appearance. Because of the functional and aesthetic implications of this condition, treatment typically requires a comprehensive approach combining orthodontics and orthognathic surgery. Surgical correction may include a Le Fort I osteotomy of the maxilla and, when indicated, a bilateral sagittal split osteotomy (BSSO) of the mandible. The goals of this type of surgery are to create a long-term stable occlusal relationship, restore facial harmony, and improve overall function (1–3).

Traditional surgical approaches to model transfer remain the most commonly used methods for transferring a patient's preoperative plan to the operating field. The traditional approach includes a facebow transfer, articulator-mounted dental models, and fabrication of intermediate and final occlusal wafers. These wafer components assist surgeons in positioning both the maxilla and the mandible during surgery. While the traditional methods have demonstrated success in achieving accurate occlusions, they require significant time and technical skill. Each step of this process may add errors due to manual adaptation of the wafers to the specific reference lines created, distortion resulting from bending of plates, and creation of inaccurate reference lines. These errors can increase the amount of time required to complete surgery, create challenges in the management of patients within the operative environment, and potentially impede postoperative recovery (4–6). There are documented accounts of clinical outcomes following traditional approaches that demonstrate satisfactory results in terms of achieved occlusion. However, variability remains in the efficiency of operations and the quality of postsurgical recovery (7–9).

The use of computer-guided orthognathic surgery, incorporating CAD/CAM-generated cutting guides, hole-locating systems, and pre-bent fixation plates, may provide a more structured method of transferring the surgical plan intraoperatively. Previous studies have suggested that CAD/CAM-assisted planning may facilitate operative workflow, shorten operative time, reduce blood loss, and simplify fixation of bone segments (10–14).

Additionally, several recent systematic reviews have shown that computer-guided and fully guided orthognathic techniques may improve reproducibility and consistency within the surgical workflow and may contribute to favorable clinical outcomes (15–18). Other studies evaluating perioperative outcomes have reported reduced operative complexity and practical advantages associated with CAD/CAM-assisted treatment planning (19–23). While many published studies have emphasized technical precision and three-dimensional positional accuracy, fewer have evaluated clinically relevant outcomes such as operative time, blood loss, intraoperative and postoperative complications, wound healing, and occlusion. Understanding how computer-assisted orthognathic workflows influence perioperative clinical outcomes is therefore important when evaluating their role in routine surgical care (4, 14, 24–27). Furthermore, evidence regarding relapse, stability, and longer-term functional outcomes following computer-guided orthognathic surgery remains limited, making comprehensive evaluation of perioperative performance important (7–9, 27, 28).

### 1.1 Research questions

1. Does computer-guided plan transfer improve intraoperative handling and efficiency compared to conventional model-surgery plan transfer in patients with skeletal anterior open bite?
2. Are there differences in operative time, blood loss, and perioperative complications between computer-guided and conventional plan-transfer workflows?
3. Do computer-guided and conventional plan-transfer workflows differ in postoperative healing and anterior open bite closure?

### 1.2 Research aim

This study aimed to evaluate and compare the clinical and perioperative outcomes of computer-guided and conventional plan-transfer workflows in adults undergoing orthognathic surgery for skeletal anterior open bite, with a particular focus on surgical efficiency, complication rates, postoperative recovery, and the achievement of functional occlusion. All participants underwent virtual surgical planning, and randomization determined how the established plan was transferred intraoperatively. It was hypothesized that the two plan-transfer workflows would differ in clinical and perioperative outcomes, particularly operative time, intraoperative handling, and postoperative recovery.

## 2 Materials and methods

### 2.1 Study design and setting

This prospective randomized controlled study examined differences in the clinical and perioperative parameters of computer-guided and conventional plan-transfer workflows following virtual surgical planning for the treatment of skeletal anterior open bite. The study received the necessary approval from the Institutional Review Board (IRB) of the Faculty of Dentistry, Al-Quds University, Palestine, where the study was conducted. Specifically, Institutional Review Board approval was obtained (approval number IRB-OMFS-2025-057; approval date: July 22, 2025); additionally, the study adhered to the ethical guidelines of the Declaration of Helsinki (2013 revision). All participants provided written informed consent before enrollment in the study.

### 2.2 Study population

A total of eighteen adult patients diagnosed with severe skeletal anterior open bite were enrolled in this study. The participants included eight males and ten females. The overall mean age was 21.7 years, with group means of 22.0 ± 3.7 years in the computer-guided group and 21.3 ± 3.0 years in the conventional group. All participants were aged ≥18 years, with an age range of 18–29 years.

Participants were randomly allocated to either the computer-guided surgery group (*n* = 9) or the conventional model surgery group (*n* = 9). The allocation sequence was generated using computer-generated random numbers by an independent research assistant who was not involved in patient enrollment or surgical procedures. Treatment assignments were concealed in closed envelopes prepared and held by the departmental research coordinator, who was not involved in clinical assessments. The allocation envelope was opened after enrollment and completion of baseline assessments and before commencement of group-specific preoperative preparation, thereby allowing sufficient time for guide design, three-dimensional printing, and plate pre-bending in participants assigned to the computer-guided group.

Blinding of the operating surgical team was not feasible because of the evident differences between the two surgical workflows. Participants were not blinded to group allocation. Postoperative outcome assessors were blinded to treatment allocation, and the statistical analyst remained blinded to group allocation until completion of the final analysis.

Adults were eligible for inclusion if they were at least 18 years of age, exhibited a skeletal anterior open bite due to posterior vertical maxillary excess, mandibular deficiency, or a combination thereof, and did not have craniofacial abnormalities or temporomandibular joint disorders. Subjects were excluded if they presented with a primarily dental open bite without a skeletal open bite, had systemic conditions that could interfere with wound healing, had undergone previous orthognathic surgery, had deformity resulting from trauma or tumors, had uncontrolled diabetes, or could not be expected to comply with treatment.

### 2.3 Sample size

No formal *a priori* sample-size calculation was performed. The sample consisted of eligible adult patients meeting the predefined selection criteria during the recruitment period, and the study was therefore considered exploratory in nature. Consequently, the relatively small sample limits statistical power to detect modest between-group differences, and the findings should be interpreted as preliminary and confirmed in larger adequately powered trials.

### 2.4 Virtual surgical planning and plan-transfer workflows

All patients underwent preoperative clinical, photographic, and radiographic assessments, including panoramic and lateral cephalometric radiographs, as well as multislice computed tomography (CT) of the craniofacial region. Dental impressions were taken to fabricate study casts representing the preoperative occlusion.

Before allocation, all enrolled patients underwent three-dimensional surgical planning and virtual plan verification to establish the definitive skeletal and occlusal treatment plan. Randomization therefore primarily determined the method by which the established plan was transferred and executed intraoperatively: computer-guided transfer or conventional model-surgery transfer.

For patients in the computer-guided surgery group, a maxillary vacuum stent with embedded radiopaque markers was used to capture the maxillomandibular relationship in centric relation during CT acquisition. The resulting CT data in DICOM format were merged with high-resolution digital dental scans to generate composite craniofacial models. Virtual osteotomies, including a Le Fort I maxillary osteotomy and, when indicated, bilateral sagittal split osteotomy (BSSO) of the mandible, were planned with the jaws positioned according to the intended final occlusal relationship. Computer-aided design/computer-aided manufacturing (CAD/CAM) cutting guides, locating-hole guides, and pre-bent fixation plates were then fabricated to facilitate accurate transfer of the virtual surgical plan to the operating room.

Following the shared virtual surgical planning phase, the computer-guided plan-transfer workflow comprised fabrication of a three-dimensional printed model, design and fabrication of cutting and locating-hole guides, preoperative adaptation of fixation plates, and intraoperative transfer of the planned osteotomy and fixation positions using patient-specific guides and predetermined screw holes (10, 13, 17, 22, 26).

During virtual simulation, maxillary transverse position, roll, and yaw were corrected according to the individual dentofacial deformity. Maxillary repositioning was planned according to the intended occlusal-plane orientation and maxillary incisor–upper lip relationship. The planned maxillary movements averaged 3.5 ± 1.2 mm of impaction and 2.0 ± 0.8 mm of advancement. In patients undergoing BSSO with mandibular advancement, the planned advancement averaged 6.0 ± 1.5 mm, with counterclockwise rotational correction performed when clinically indicated. In patients undergoing genioplasty, mean advancement was 4.0 ± 1.0 mm.

For patients in the conventional model surgery group, dental casts were mounted on an articulator using a facebow transfer. Intermediate and final occlusal wafers were fabricated to guide intraoperative positioning of the maxilla and mandible. Internal and external reference points were used to verify vertical and horizontal alignment throughout the surgical procedure.

Following the shared virtual surgical planning phase, the conventional plan-transfer workflow involved facebow transfer, articulator mounting, predictive model surgery for transfer of the predefined movements, fabrication of intermediate and final occlusal wafers, and intraoperative verification of maxillary position using internal and external reference measurements. Unlike the computer-guided workflow, fixation plates were manually adapted intraoperatively. Thus, the computer-guided approach transferred a greater proportion of plan-transfer and fixation preparation to the preoperative digital phase, whereas the conventional approach required greater manual transfer and adaptation during surgery.

### 2.5 Procedure overview

All surgical procedures were performed under general anesthesia with nasotracheal intubation. A Le Fort I osteotomy was performed to reposition the maxilla, and, when indicated, a bilateral sagittal split osteotomy (BSSO) was performed to reposition the mandible. In the computer-guided group, the cutting guide was positioned on the exposed maxillary surface to transfer the planned osteotomy level, while the locating-hole guide determined the predetermined screw positions. Following maxillary mobilization and removal of bony interferences, the pre-bent fixation plates were secured through the predetermined holes. The final occlusal wafer was subsequently used to guide mandibular positioning when BSSO was performed.

In contrast, in the conventional model surgery group, the maxilla and mandible were moved into their new positions using the occlusal wafers created preoperatively; the titanium fixation plates were bent and adapted intraoperatively. Routine postoperative soft-tissue care protocols were followed for all patients. The specific combination of osteotomies and adjunctive procedures was individualized according to each patient's dentofacial deformity and clinical treatment requirements. Randomization determined the plan-transfer workflow rather than the clinically indicated skeletal procedure.

### 2.6 Outcome measures

The primary outcomes were intraoperative handling, operative time, and intraoperative blood loss. Intraoperative handling was a descriptive subjective outcome assessed by the operating surgical team on the basis of ease of transferring the planned jaw position, the requirement for repeated positional verification, the need for intraoperative segment readjustment, and the extent of fixation-plate adaptation. No validated numerical or ordinal handling scale was used; therefore, intraoperative handling was not subjected to inferential statistical testing.

Secondary outcomes included postoperative wound healing, postoperative complications, including hardware loosening and malocclusion, anterior open bite closure measured clinically using a caliper, achievement of the planned occlusion, and the need for postoperative orthodontic adjustment.

Postoperative clinical follow-up was performed at 1 week, 2 weeks, 1 month, 3 months, and 6 months. Postoperative edema was assessed clinically at each scheduled follow-up examination and was considered resolved at the first follow-up visit at which facial and intraoral swelling was no longer clinically evident. Because assessments were performed at discrete follow-up intervals, the exact time of edema resolution was interval-censored. Accordingly, time to clinical edema resolution was summarized using the median and interquartile range (IQR). Edema severity was not assessed using volumetric measurements or a validated severity scale.

### 2.7 Statistical analysis

Continuous variables are presented as mean ± standard deviation (SD) when appropriate, whereas time to clinical edema resolution is presented as median and interquartile range (IQR) because resolution was assessed at discrete follow-up intervals. Categorical variables are presented as frequencies. Between-group comparisons of continuous variables were performed using the independent-samples *t*-test or an appropriate nonparametric test when the data were not normally distributed. Time to clinical edema resolution was summarized descriptively using the median and IQR because the exact resolution time could not be determined between scheduled follow-up examinations. Categorical baseline characteristics were summarized descriptively. Fisher's exact test was used for categorical outcome comparisons when inferential analysis was applicable because of the small expected cell frequencies. Statistical significance was set at *p* < 0.05. All statistical analyses were performed using IBM SPSS Statistics version 20 (IBM Corp., Armonk, NY, USA).

## 3 Results

### 3.1 Patient characteristics and surgical procedures

A total of 18 participants completed the study, with no withdrawals during the follow-up period. Baseline demographic and clinical characteristics are summarized in Table 1. Each group included four males and five females. The mean age was 22.0 ± 3.7 years in the computer-guided group and 21.3 ± 3.0 years in the conventional model surgery group.

**Table 1:** Patient characteristics and surgical procedures.

Parameter	Computer-guided group (*n* = 9)	Conventional group (*n* = 9)
Age, mean ± SD (years)	22.0 ± 3.7	21.3 ± 3.0
Sex (M/F)	4/5	4/5
Preoperative AOB, mean ± SD (mm)	9.50 ± 3.64	8.09 ± 2.11
Le Fort I only	1 (11.1%)	1 (11.1%)
Le Fort I + BSSO	8 (88.9%)	8 (88.9%)
Genioplasty	3 (33.3%)	2 (22.2%)
Septoplasty	1 (11.1%)	1 (11.1%)
Skeletal Class I	2 (22.2%)	3 (33.3%)
Skeletal Class II	4 (44.4%)	3 (33.3%)
Skeletal Class III	3 (33.3%)	3 (33.3%)
Posterior vertical maxillary excess	6 (66.7%)	5 (55.6%)
Mandibular deficiency/short ramus	7 (77.8%)	6 (66.7%)
Combined skeletal deformity	4 (44.4%)	3 (33.3%)

Table 1 provides an expanded baseline clinical profile and distribution of surgical procedures. One patient in each group underwent Le Fort I osteotomy without BSSO, whereas eight patients in each group underwent combined Le Fort I + BSSO. Adjunctive procedures were individualized according to clinical requirements.

### 3.2 Intraoperative findings and perioperative outcomes

All operative procedures were completed successfully, and there were no significant intraoperative complications. Intraoperative management was reportedly easier from the perspective of the surgical team in the computer-guided group, since the cutting and locating-hole guides provided direct transfer of the planned maxillary osteotomy and fixation locations, and the pre-bent plates eliminated the need for intraoperative plate adaptation. The traditional method utilized multiple verifications via both internal and external references and intraoperative plate adaptation. Because handling was not measured quantitatively using a validated tool, this finding is reported as a subjective observation of an observed difference rather than a tested treatment outcome.

The mean operative time was 6.0 ± 0.5 h in the computer-guided group and 6.5 ± 0.6 h in the conventional model surgery group, although the difference was not statistically significant (*p* = 0.08). Accordingly, the observed 0.5-hour difference represents a nonsignificant numerical trend rather than evidence of a statistically demonstrated reduction in operative time.

Because operative duration may vary according to procedural complexity, operative times were also examined by procedure type. The single Le Fort I-only case required 5.0 h in the computer-guided group and 5.5 h in the conventional group; these single-case observations were descriptive and were not statistically compared. Among the eight patients per group undergoing Le Fort I + BSSO, mean operative time was 6.1 ± 0.4 h in the computer-guided group and 6.6 ± 0.5 h in the conventional group. Among procedures that also included genioplasty, mean operative time was 6.5 ± 0.3 h and 7.0 ± 0.4 h, respectively. These stratified findings were interpreted descriptively because of the small subgroup sizes.

**Figure 1:**
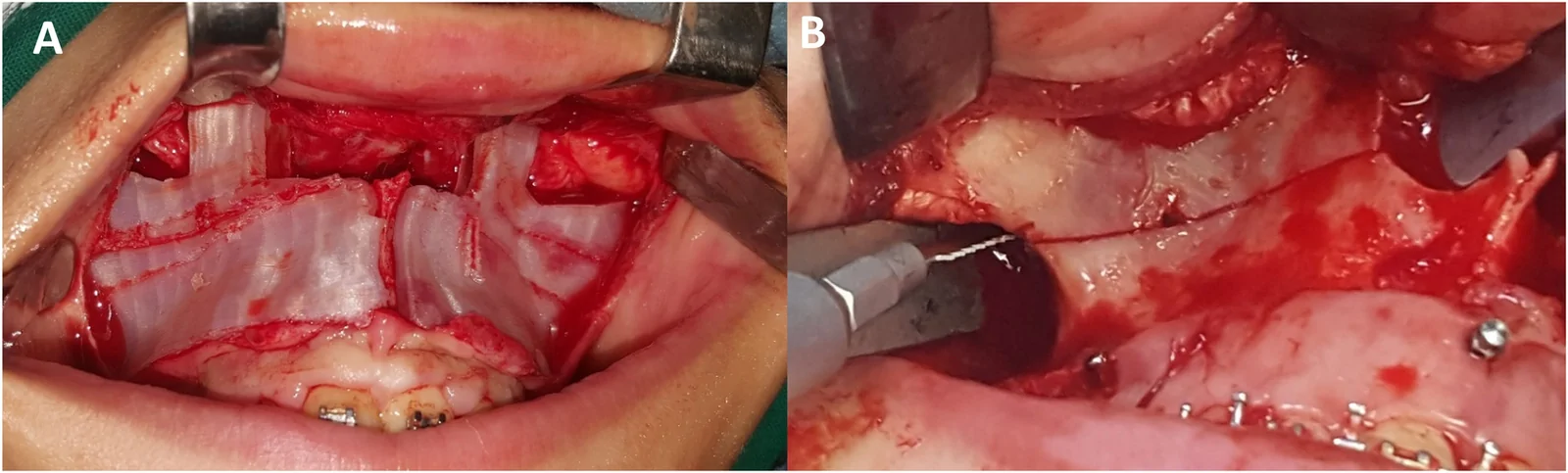
**(A)** Preoperative frontal and lateral clinical photographs at rest and smiling. **(B)** Corresponding postoperative frontal and lateral photographs at rest and smiling following conventional model surgery. Panels **(A,B)** are identified within the figure, and the eye region is masked to protect participant privacy.

Mean intraoperative blood loss was 450 ± 60 mL in the computer-guided group and 520 ± 80 mL in the conventional group, with no statistically significant difference (*p* = 0.12). No uncontrolled bleeding or clinically significant displacement of osteotomy segments occurred. Transient postoperative paresthesia was observed, but no persistent sensory deficit was identified during follow-up (Table 2; Figure 1).

**Table 2:** Clinical and perioperative outcomes in the computer-guided and conventional plan-transfer groups.

Outcome	Computer-guided group	Conventional group	*p*-value
Mean operative time (hours)	6.0 ± 0.5	6.5 ± 0.6	0.08
Blood loss (mL)	450 ± 60	520 ± 80	0.12
Intraoperative complications	0	0	N/A
Postoperative complications requiring clinical intervention	2 patients	1 patient	N/A
Time to clinical edema resolution, median (IQR), weeks	2 (1–2)	2 (1–2)	N/A
Transient paresthesia	9 (100%)	9 (100%)	N/A

### 3.3 Postoperative healing and complications

Overall postoperative recovery was favorable in both groups, with no wound dehiscence, infection, plate exposure, or other major wound-healing complications. Postoperative swelling was assessed clinically during scheduled follow-up. Median time to clinical edema resolution was 2 weeks (IQR: 1–2) in the computer-guided group and 2 weeks (IQR: 1–2) in the conventional group. Because edema resolution was determined only at scheduled follow-up examinations, these values represent the first visit at which edema was no longer clinically evident rather than the exact biological time of resolution. Because edema severity was not quantified using an objective scale or volumetric measurement, no formal between-group comparison of edema severity was performed.

Transient postoperative paresthesia was reported in all patients and resolved within one month; no persistent sensory deficit was observed. Three postoperative complications requiring intervention were recorded. In the computer-guided group, one patient developed transient midline displacement with a residual anterior open bite that was managed with guiding elastics, while another developed loosening of a mandibular fixation screw and underwent reoperation for hardware repositioning. In the conventional group, one patient developed mild malocclusion secondary to plate loosening, which was managed by local plate adjustment and short-term guiding elastics. All three complications resolved without persistent sequelae during follow-up.

### 3.4 Anterior open bite closure and occlusal outcomes

Both groups showed substantial improvement in the anterior open bite; there were no statistically significant differences in early postoperative AOB measurements between the two groups. The mean preoperative anterior open bite dimensions were 9.50 ± 3.64 mm and 8.09 ± 2.11 mm for the computer-guided and conventional groups, respectively (*p* = 0.33), while the mean postoperative values were 1.33 ± 0.50 mm in both groups (*p* = 1.00).

**Figure 2:**
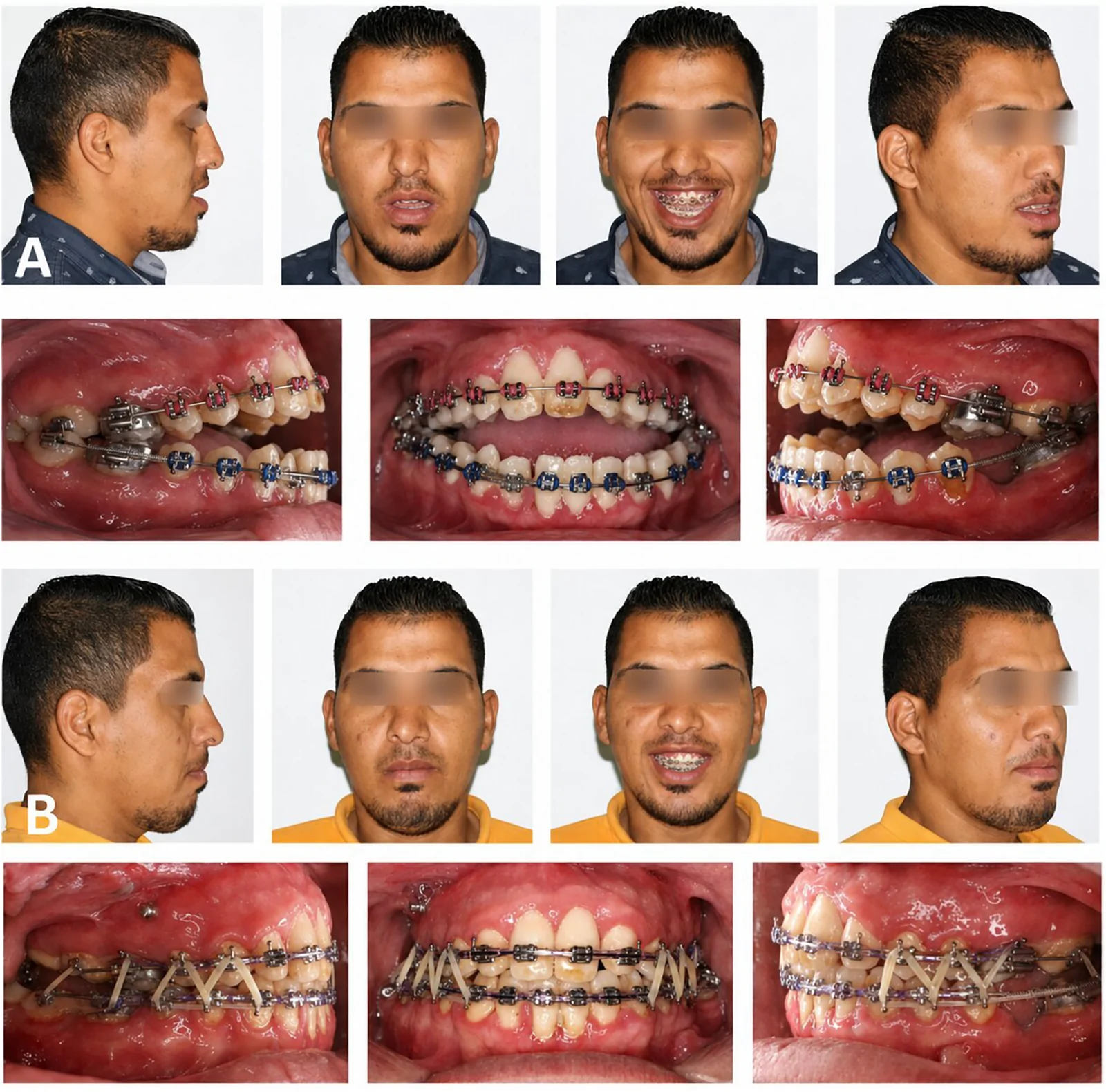
**(A)** Preoperative frontal clinical photographs at rest and smiling. **(B)** Corresponding postoperative frontal photographs at rest and smiling following computer-guided orthognathic surgery. The eye region is masked to protect participant privacy.

**Figure 3:**
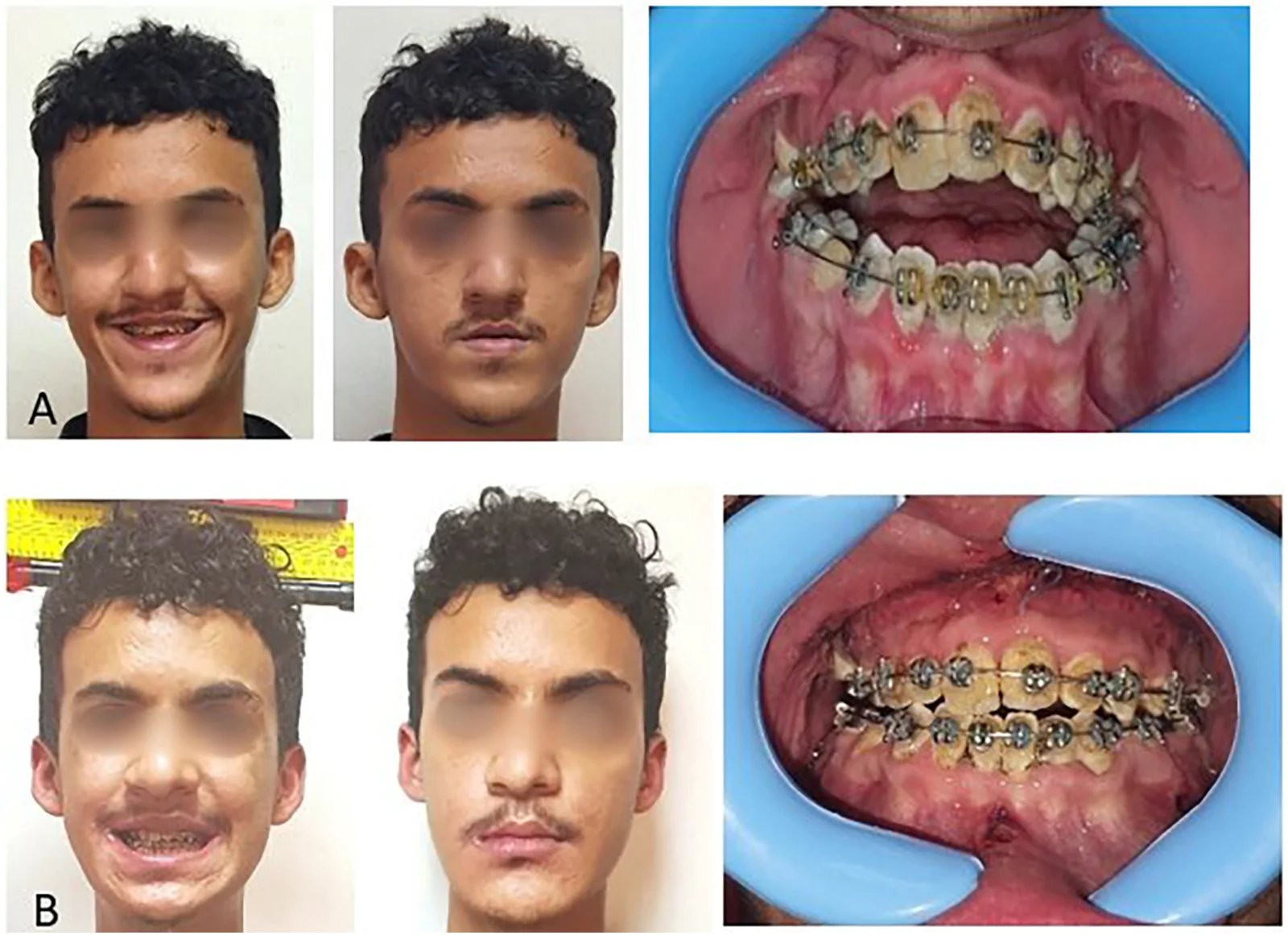
Intraoperative comparison of computer-guided and conventional techniques during Le fort I osteotomy. **(A)** Application of a CAD/CAM-generated cutting guide for osteotomy positioning in the computer-guided group. **(B)** Conventional freehand osteotomy positioning using manual instrumentation and intraoperative reference measurements in the conventional model surgery group.

After postoperative management of all patients, adequate functional occlusion was established. In one case from the computer-guided group, there was initially an anterior open bite and a transverse shift of the midline, which were corrected using guiding elastics. As part of standard care, minor orthodontic adjustments were performed, if necessary, after completion of postoperative management. Figures 2, 3 show representative pre- and postoperative photographs illustrating the correction of the anterior open bite and the establishment of a functionally acceptable occlusion (Table 3).

**Table 3:** Preoperative and early postoperative anterior open bite measurements in the computer-guided and conventional plan-transfer groups.

Parameter	Study group (mm)	Control group (mm)	*p*-value
Preoperative AOB	9.50 ± 3.64	8.09 ± 2.11	0.33
Postoperative AOB	1.33 ± 0.50	1.33 ± 0.50	1.00

## 4 Discussion

This study compared the clinical and perioperative outcomes of computer-guided and conventional plan-transfer workflows following virtual surgical planning for skeletal anterior open bite. No statistically significant between-group differences were detected in operative time, blood loss, or early postoperative anterior open bite. Given the small sample size and limited statistical power, these null findings should not be interpreted as demonstrating equivalence between the two plan-transfer workflows. However, the operating surgical team noted that the computer-guided method facilitated intraoperative plan transfer and handling. Because this observation was subjective, it cannot be concluded from these comments that one method has a clinical advantage over the other.

The surgical team perceived practical advantages in transferring the operative plan when utilizing the computer-guided methodology. These advantages included the use of CAD/CAM cutting guides and locating-hole guides to transfer the planned osteotomy and fixation positions, as well as the use of pre-bent fixation plates, which decreased the need for manual intraoperative plate adaptation. In comparison, the conventional model-surgery methodology utilized occlusal wafers along with internal and external references and multiple positional verifications for intraoperative plate adaptation (11, 12, 29–31). Since intraoperative handling was not assessed using an objective quantitative tool, these statements reflect descriptive information regarding the two methodologies used rather than objective statistical comparative data.

These potential intraoperative advantages must be balanced against the additional preoperative technical requirements associated specifically with computer-guided plan transfer. Although virtual surgical planning was performed for participants in both groups, the computer-guided workflow additionally required design and fabrication of patient-specific cutting and locating-hole guides, three-dimensional printing, and preoperative adaptation of fixation components. These additional steps may require greater technical expertise, digital infrastructure, and financial resources than conventional model-surgery plan transfer (5, 13, 14, 21). Preoperative preparation time and direct costs were not systematically recorded in the present study; therefore, no quantitative conclusion can be drawn regarding total workflow time or cost efficiency.

The postoperative outcome was favorable in both groups. No major wound-healing complication occurred, although three postoperative complications requiring clinical intervention were observed. These events consisted of transient malocclusion or midline/open-bite discrepancy and fixation-hardware loosening, and all were successfully managed without persistent sequelae during follow-up. Edema resolved during the early postoperative period, and paresthesia resolved within one month in all affected patients. These findings are consistent with prior clinical studies reporting generally low rates of major complications when appropriate surgical and postoperative care is provided (6, 18, 19, 22).

Substantial AOB correction was achieved in both groups. Mean preoperative AOB was 9.50 ± 3.64 mm in the computer-guided group and 8.09 ± 2.11 mm in the conventional group, whereas mean early postoperative AOB was 1.33 ± 0.50 mm in both groups. Substantial short-term occlusal correction was observed in both groups; however, the present study was not designed or powered to demonstrate equivalence between the two plan-transfer workflows. The identical postoperative group means do not establish equivalence or superiority of either technique for AOB closure.

The quantitative perioperative findings likewise require cautious interpretation. The computer-guided group showed a 0.5-hour lower mean operative time and a 70-mL lower mean blood loss; however, neither difference reached statistical significance (*p* = 0.08 and *p* = 0.12, respectively). These results therefore represent numerical trends rather than demonstrated treatment effects. Procedure-specific analysis also showed numerically shorter operative times with computer guidance, but the Le Fort I-only subgroup consisted of only one patient per group and the remaining subgroups were small, precluding robust procedure-specific inferential conclusions.

This study provides new clinical data on perioperative care to an existing literature that has focused primarily on technical and spatial (three-dimensional) accuracy outcomes (10, 16, 17, 24, 25). The results indicate that computer-guided transfer of an established virtual surgical plan may facilitate selected intraoperative steps; however, this trial found no statistically significant differences in the primary quantitative perioperative outcomes compared with conventional plan transfer.

Each surgery was tailored based on the unique skeletal deformities of the patients involved instead of being standardized for research purposes. In addition, one patient from each treatment group underwent a Le Fort I osteotomy alone, while eight patients from each treatment group underwent both a Le Fort I osteotomy and a bilateral sagittal split osteotomy (BSSO); three patients who received computer-guided treatment underwent genioplasty, while two patients who received conventional treatment also underwent genioplasty. Although the distributions of major procedures between the treatment groups were similar, it is possible that variations in the severity of individual deformities, as well as variability in adjunctive procedures performed during surgery, could influence operative time and/or blood loss. Therefore, when comparing perioperative outcomes, case-mix variation must be taken into account.

There are several limitations associated with this study. First, a formal *a priori* sample-size determination was not conducted, thus limiting the ability to detect statistically significant differences between groups if such differences exist. Second, subjective assessments were used to evaluate intraoperative management and therefore lacked validation through the use of a reliable objective tool. Operative time and blood loss represent objective quantitative measurements. Third, the extent of individual skeletal movement and adjunctive procedures was determined by the need to address specific clinical issues and therefore resulted in variable degrees of complexity in procedural implementation. Fourth, because preoperative preparation time and financial costs were not evaluated systematically, direct comparisons between total workflows and cost efficiencies cannot be made. Finally, patients were followed postoperatively, but the overall long-term assessment of skeletal stability, relapse and patient-reported functional outcomes was not addressed.

These limitations can be addressed in future studies through the use of quantitative intraoperative measures, larger sample sizes achieved through multicenter recruitment, and longer follow-up periods to achieve a better understanding of both the clinical and functional implications of computer-assisted orthognathic surgery (7–9, 27, 28).

Overall, no statistically significant differences were detected between the computer-guided and conventional plan-transfer workflows in operative time, intraoperative blood loss, or AOB during the initial weeks after surgery. Although computer-guided plan transfer was perceived to facilitate intraoperative plan transfer and management, this observation was subjective and therefore should be interpreted cautiously. Given the small sample size and limited statistical power, the absence of statistically significant differences should not be interpreted as evidence that the two workflows are equivalent. Computer-guided transfer may offer practical advantages during selected intraoperative steps, but these observations require confirmation using objective measures in larger adequately powered studies.

## 5 Conclusion

This randomized controlled trial detected no statistically significant differences between computer-guided and conventional plan-transfer workflows in operative time, intraoperative blood loss, or postoperative AOB among adults undergoing orthognathic surgery for skeletal anterior open bite. Because of the small sample size and limited statistical power, these findings should not be interpreted as demonstrating equivalence between the two workflows.

The principal contribution of this study is the provision of randomized clinical evidence extending previous accuracy-focused research to clinically relevant perioperative outcomes, including operative efficiency, complications, postoperative recovery, and occlusal correction. Although computer-guided plan transfer was perceived to facilitate intraoperative handling and showed a nonsignificant trend toward shorter operative time, these findings do not establish superiority or equivalence compared with conventional plan transfer. Larger adequately powered studies incorporating objective workflow measures, assessment of preoperative resource requirements, procedure-specific analyses, and longer-term skeletal and functional follow-up are needed to determine the clinical significance of these potential workflow advantages.

## Data Availability

The raw data supporting the conclusions of this article will be made available by the authors, without undue reservation.
